# The role of the cerebellum in degenerative ataxias and essential tremor: Insights from noninvasive modulation of cerebellar activity

**DOI:** 10.1002/mds.27919

**Published:** 2019-12-10

**Authors:** Roderick P.P.W.M. Maas, Rick C.G. Helmich, Bart P.C. van de Warrenburg

**Affiliations:** ^1^ Department of Neurology & Donders Institute for Brain, Cognition, and Behaviour Radboud University Medical Center Nijmegen the Netherlands

**Keywords:** degenerative cerebellar ataxia, essential tremor, noninvasive cerebellar stimulation, transcranial direct current stimulation, transcranial magnetic stimulation

## Abstract

Over the last three decades, measuring and modulating cerebellar activity and its connectivity with other brain regions has become an emerging research topic in clinical neuroscience. The most important connection is the cerebellothalamocortical pathway, which can be functionally interrogated using a paired‐pulse transcranial magnetic stimulation paradigm. Cerebellar brain inhibition reflects the magnitude of suppression of motor cortex excitability after stimulating the contralateral cerebellar hemisphere and therefore represents a neurophysiological marker of the integrity of the efferent cerebellar tract. Observations that cerebellar noninvasive stimulation techniques enhanced performance of certain motor and cognitive tasks in healthy individuals have inspired attempts to modulate cerebellar activity and connectivity in patients with cerebellar diseases in order to achieve clinical benefit. We here comprehensively explore the therapeutic potential of these techniques in two movement disorders characterized by prominent cerebellar involvement, namely the degenerative ataxias and essential tremor. The article aims to illustrate the (patho)physiological insights obtained from these studies and how these translate into clinical practice, where possible by addressing the association with cerebellar brain inhibition. Finally, possible explanations for some discordant interstudy findings, shortcomings in our current understanding, and recommendations for future research will be provided. © 2019 The Authors. *Movement Disorders* published by Wiley Periodicals, Inc. on behalf of International Parkinson and Movement Disorder Society.

The cerebellum is a highly complex brain region that fulfills a crucially important role in a variety of seemingly natural processes, including postural control, locomotion, and numerous cognitive functions. As an integration center receiving multimodal sensorimotor information from the spinal cord, cerebral cortex, and vestibular nuclei, it continuously compares efference copies and reafference signals and corrects for discrepancies between them to enable the execution of smooth, well‐coordinated movements. Notably, given the dearth of direct connections between the cerebellum and peripheral nervous system, this intricate task is mainly accomplished by modulating the excitability of the primary motor cortex through the cerebellothalamocortical tract.^1^, ^2^

In 1995, Ugawa and colleagues demonstrated the possibility of quantifying the integrity of the cerebellothalamocortical pathway as a neurophysiological outcome measure by means of a painless paired‐pulse transcranial magnetic stimulation (TMS) paradigm.^3^, ^4^ In an influential series of experiments, they showed a reduction of the motor evoked potential (MEP) amplitude when a conditioning stimulus was delivered across the contralateral skull base in a time window of 5 to 7 ms prior to a magnetic pulse over the motor cortex.^4^ Given the absence of such a suppression effect in two patients with cerebellar dysfunction and the observation that the most effective stimulation position to diminish MEP amplitude corresponded to the cerebellar hemisphere, they deduced that the cerebellum must play a pivotal role. From a physiological perspective, Purkinje cells exert an inhibitory tone on the deep cerebellar nuclei, which constitute the sole cerebellar outflow system. Their excitatory projections to the contralateral motor cortex are relayed within the ventrolateral thalamus (Fig. 1). The net effect of a cerebellar conditioning stimulus would therefore consist of an activation of Purkinje cells, inhibition of deep cerebellar nuclei, and thus a reduction of the contralateral motor cortex excitability, a phenomenon called cerebellar brain inhibition (CBI). Subsequent investigations in patients with cerebellar ataxia due to lesions in different pathways revealed that CBI is reduced or absent in case of pathology involving the efferent cerebellar tract. On the other hand, when afferent cerebellar systems were implicated, patients were found to display normal motor cortex inhibition.^5^, ^6^, ^7^, ^8^

**Figure 1:**
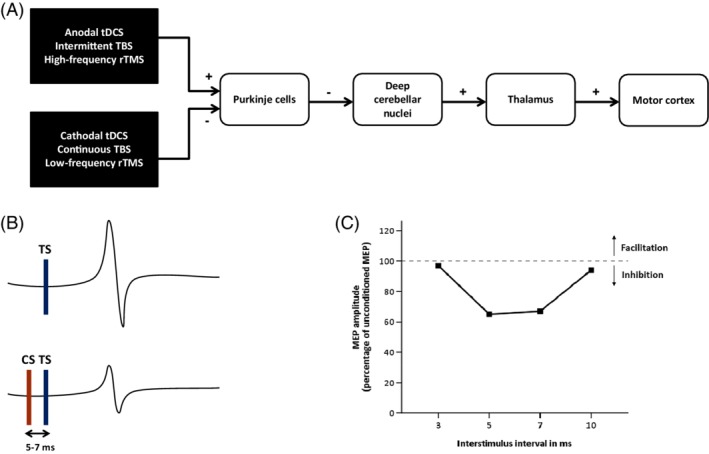
**(A)** Excitatory and inhibitory projections of the cerebellothalamocortical pathway and the putative effects of cerebellar anodal and cathodal transcranial direct current stimulation (tDCS), high‐frequency and low‐frequency repetitive transcranial magnetic stimulation (rTMS), and intermittent and continuous theta burst stimulation (TBS). **(B)** A single suprathreshold TMS pulse over the primary motor cortex elicits a motor evoked potential (MEP), which decreases in size when the test stimulus (TS) is preceded by a conditioning stimulus (CS) over the contralateral cerebellar hemisphere within an interval of 5 to 7 ms. This physiological phenomenon, which depends on the integrity of the cerebellothalamocortical pathway, is called cerebellar brain inhibition (CBI). **(C)** The MEP amplitude following paired‐pulse stimulation is expressed as a percentage of the unconditioned MEP amplitude. Significant suppression in healthy individuals typically occurs when interstimulus intervals are set at 5 to 7 ms. [Color figure can be viewed at http://wileyonlinelibrary.com]

In healthy individuals, repeated CBI measurements have been conducted to examine the role of the cerebellum and dynamic alterations of its excitability in motor tasks. These studies have contributed to a better understanding of the underlying neural basis of motor learning. For instance, the decline of CBI that was found to occur in a visuomotor reach adaptation task when perturbations were introduced abruptly (large errors), but not gradually (small errors), suggests that distinct neural mechanisms are engaged in response to errors of varying size and that the cerebellum is most implicated early on during the motor adaptation process.^9^ A reduction of CBI magnitude was later confirmed to be specific to the early (rather than late) stage of skill learning.^10^, ^11^ Interestingly, CBI alterations correlated with the amount of skill acquisition and locomotor adaptation, which indicates a direct association between changes in the degree of cerebellar excitability and performance of these tasks.^10^, ^12^ Similar reductions in CBI have been reported during both the execution and observation of a visuomotor procedural learning task that is dependent on a proper cerebellar function, but only if the learning had not been acquired previously.^13^ The mechanistic underpinning of reduced CBI during the process of motor learning possibly entails a temporarily diminished excitability of Purkinje cells due to a decrease in synaptic transmission efficacy between parallel fibers and Purkinje cells, emanating from simultaneous activation of climbing and parallel fibers. This phenomenon has been established in vivo in animal studies and named long‐term depression.^11^, ^14^, ^15^, ^16^, ^17^

Beyond measuring the excitability of the cerebellum, modulation of its activity and connectivity with other brain regions by noninvasive stimulation techniques has gained increasing attention and may represent a novel exciting approach in the treatment of cerebellar disorders. In this article, after an introduction highlighting some basic physiological principles and promising study results in healthy individuals, we comprehensively explore the therapeutic potential of noninvasively modulating a damaged and/or malfunctioning cerebellum and show how this has expanded our pathophysiological insights. Where possible, the association with CBI will be addressed. Finally, possible explanations for the sometimes discordant interstudy findings, shortcomings in our current understanding, and recommendations for future research will be discussed.

## Noninvasive Modulation of Cerebellar Activity in Healthy Adults

Following the widespread usage of transcranial direct current stimulation (tDCS) and repetitive TMS (rTMS), including theta burst stimulation (TBS) protocols, over different areas of the cerebral cortex, the similar application of these techniques over the cerebellum has only relatively recently been embraced. Both modalities have been shown to induce changes in neural activity that outlast the duration of stimulation. More specifically, low‐frequency rTMS (≤ 1 Hz), continuous TBS (cTBS), and cathodal tDCS decrease the excitability of neurons in the targeted region, whereas opposite effects can be elicited by high‐frequency rTMS (≥ 5 Hz), intermittent TBS (iTBS), and anodal tDCS.^18^ Although the net results of these interventions with respect to neuronal excitability may be similar, each of them has intrinsic advantages and disadvantages that have been discussed extensively elsewhere.^19^

Trains of repetitive magnetic stimuli generally provoke a transient modulation of neural activity in a selected cortical area, which may elicit measurable behavioral effects. Low‐frequency rTMS over the medial cerebellum in healthy adults has been demonstrated to reduce the magnitude of saccadic adaptation and increase variability on the paced finger‐tapping task, whereas an impairment of procedural learning was observed when the cerebellar hemispheres were targeted.^20^, ^21^, ^22^ Furthermore, application of cTBS over the right cerebellar hemisphere interfered with the normal acquisition of conditioned eyeblink responses in individuals naïve to this motor learning paradigm and brought about a lower number of category switches during the early phase of a phonemic fluency task.^23^, ^24^ Last, cTBS delivered over the midline cerebellum increased the sway path length and the oscillations of the center of pressure.^25^ Long‐term depression‐like effects are proposed to underlie the aforementioned behavioral manifestations of cerebellar cTBS and low‐frequency rTMS.^18^ Indeed, observed neurophysiological alterations directly following the administration of these interventions in healthy adults include an increased unconditioned MEP amplitude derived from contralateral motor cortex stimulation and a significant CBI reduction that lasted for at least 30 minutes after the end of stimulation.^26^, ^27^ These changes probably indicate the release of tonic inhibition of Purkinje cells on deep cerebellar nuclei. High‐frequency rTMS and iTBS, on the other hand, are thought to increase synaptic strength (long‐term potentiation‐like effects).^18^ Accordingly, midline cerebellar iTBS targeting one of the nodes of the dorsal attentional network was found to enhance functional connectivity with cortical regions within this network and improved sustained attentional control, while an induced alteration of the activity in the posterolateral cerebellum, more specifically Crus I/II, modulated the connectivity within the default mode network.^28^, ^29^

At variance with TMS, which employs the principle of electromagnetic induction and involves the use of an external coil to instantaneously generate action potentials in cortical axons, the weak electric currents (1–2 mA) that are applied in tDCS by means of two electrodes are thought to modulate the excitability of the neuronal membrane potential toward depolarization (anodal stimulation) or hyperpolarization (cathodal stimulation).^30^ The orientation of neural structures in the target area relative to the current flow and their pre‐existing physiological state are two key factors that critically affect the likelihood of neuronal discharge and therefore the efficacy of stimulation.^31^ In modeling studies, cerebellar tDCS has been shown to selectively influence the cerebellar hemispheres with only minor spread of the electric field toward the occipital cortex and negligible spreading to the brainstem and the heart, rendering this a safe technique.^32^, ^33^ In a seminal article, Galea and colleagues showed that a 25‐minute session of tDCS over the right cerebellar hemisphere can modulate cerebellar excitability in young, healthy individuals in a polarity‐specific manner– that is, anodal tDCS increased CBI, cathodal tDCS decreased CBI, and sham stimulation left CBI unchanged.^34^ Subsequently, anodal cerebellar tDCS was reported to enhance acquisition in visuomotor adaptation, locomotor adaptation, delay eyeblink conditioning, skill learning, and postural control adaptation tasks in healthy individuals.^35^, ^36^, ^37^, ^38^, ^39^ These results could, however, not always be reproduced.^40^, ^41^ Cathodal cerebellar tDCS, on the other hand, caused slower adaptation, significantly fewer conditioned responses in an eyeblink conditioning paradigm, and reduced forward digit spans.^36^, ^38^, ^42^ However, a recent meta‐analysis found no evidence for such polarity‐dependent effects.^43^

Although most investigations that evaluated the effects of cerebellar tDCS were performed in young adults, positive results on postural stability and motor learning have also been described in healthy elderly individuals.^44^, ^45^, ^46^ A single round of cerebellar anodal tDCS decreased postural sway and increased Berg Balance Scale (BBS) scores compared to sham stimulation in a randomized double‐blind study.^44^ Furthermore, while older individuals are known to display a considerably slower motor adaptation rate, it took only one bout of anodal cerebellar tDCS to acquire a performance level comparable to that of a younger sham group.^45^ Finally, as an adjunct to postural training, multiple rounds of bilateral cerebellar anodal tDCS improved anterior‐posterior and mediolateral stability indices and BBS scores in a multiple‐arm, randomized controlled trial in older adults with a high fall risk, whereas the groups that received only postural training or bilateral cerebellar anodal tDCS remained at baseline level.^46^

Besides rTMS and tDCS, transcranial alternating current stimulation (tACS) has recently emerged as a third noninvasive neuromodulation technique.^47^ In brief, externally imposed sinusoidal currents at a specific frequency, phase, and intensity selectively intervene with endogenous brain oscillations, which may, in turn, generate electrophysiological and behavioral effects.^31^, ^48^, ^49^ Indeed, the application of cerebellar tACS at a frequency near the basal firing rate of Purkinje cells (50 Hz) has been shown to modulate CBI and affect the performance of a right‐hand motor task.^50^, ^51^ Interestingly, interference with the surrounding inhibitory interneuronal network using 300‐Hz tACS induced opposite effects on CBI, highlighting the frequency specificity of this modality.^50^, ^51^ Finally, simultaneous antiphase gamma tACS over a cerebellar hemisphere and the contralateral motor cortex in order to strengthen the network may constitute a more effective strategy than isolated stimulation of each of these regions.^52^, ^53^

In summary, noninvasive cerebellar stimulation techniques are able to modulate cerebellar activity and the connectivity with other brain regions, as exemplified by the modulation of the degree of CBI. From a clinical perspective, especially cerebellar anodal tDCS has been demonstrated to enhance postural control in the elderly and induce a higher level of performance with a faster reduction of (large) errors in motor learning tasks in both younger and older adults. However, behavioral effects have not always been consistent.

## Effects of Noninvasive Cerebellar Stimulation in Diseases Affecting The Cerebellum

Following the aforementioned encouraging observations that performance of certain motor and cognitive tasks could be enhanced in healthy individuals, investigators started to explore whether modulating the excitability and connectivity of a damaged and/or malfunctioning cerebellum may also benefit patients with various types of cerebellar dysfunction.^54^ In the ensuing section, we comprehensively discuss these studies and discern two disease models that share primary involvement of the cerebellum: (1) degenerative ataxias, characterized by an insidious onset and gradually progressive course (Table 1), and (2) essential tremor (ET), which is hypothesized to arise mainly from a complex functional perturbation along the cerebellothalamocortical pathway (Table 2). Stroke‐related ataxia can be considered a third model of cerebellar dysfunction, epitomizing an acute, static cerebellar insult, but we will not review these studies in the main text of this article. A description of their results can be found in Supporting Information Table S1.

**Table 1:** Overview of studies exploring the therapeutic potential of noninvasive cerebellar stimulation techniques in patients with degenerative cerebellar ataxias

Study	Etiology (no.)	Intervention	Sham	Blinding	Protocol	Results
Shimizu, 1999^105^	SCA6 (2), SCA1 (1), SCA7 (1)	TMS over the inion and positions 4 cm left and right from the inion (circular coil)	No	N/A	10 pulses over each position every day for 21 days	Increased gait speed and fewer steps required to walk 10 m Less postural (truncal) sway and improvement of tandem gait No change in dysarthria, nystagmus, and limb ataxia
Shiga, 2002^55^	Either cerebellar cortical atrophy or OPCA (74)	TMS over the inion and positions 4 cm left and right from the inion (circular coil)	Yes	Patients and examiners	10 pulses over each position every day for 21 days	Increased gait speed (10‐m walk) and improvement of both tandem gait and standing capacities Larger beneficial effects in patients with pure cerebellar atrophy than in those with OPCA Maintained improved condition for at least 6 months if TMS was continued once or twice a week
Farzan, 2013^56^	Idiopathic late‐onset cerebellar atrophy (1)	TMS over the inion and positions 4 cm left and right from the inion (circular coil)	No	N/A	10 pulses over each position every day for 21 days	Improvements in upper limb dysmetria and tremor and speech Faster execution of the Timed Up and Go test and increased gait speed Decreased postural sway on static posturography Reduction of CBI
Grimaldi, 2013^58^	Immune (1), paraneoplastic (1), ARCA (1), ADCA (3), idiopathic adult‐onset ataxia (3)	tDCS; anode 3 cm to the right of the inion or over the inion, cathode over the contralateral supraorbital area or over the right shoulder	Yes	Patients	20 minutes stimulation, 1 or 2 mA	Lower amplitude of long‐latency stretch reflexes in the upper limbs after anodal cerebellar tDCS No clinical improvements of postural stability and upper limb coordination
Grimaldi, 2014^60^	SCA2 (2)	tCCDCS; anode 3 cm to the right of the inion, immediately followed by left motor cortex tDCS; cathode over the contralateral supraorbital area	Yes	Patients	20 minutes stimulation, 1 mA	Reduction of upper limb postural tremor (accelerometry), action tremor, and hypermetria (haptic technology) SARA score decrease of 3 and 3.5 points (no effects on gait, stance, and speech)
Benussi, 2015^64^	SCA2 (5), SCA1 (1), SCA38 (2), FA (1), AOA2 (1), MSA‐C (6), FXTAS (1), SAOA (2)	tDCS; anode over the cerebellum area, cathode over the right deltoid muscle	Yes	Patients, examiner, and outcome assessor	20 minutes stimulation, 2 mA; single session	Decrease in SARA and ICARS scores compared to sham stimulation, specifically the posture and gait and limb coordination items Increased gait speed (8MWT) and improved manual dexterity (9HPT) compared to sham stimulation
Bodranghien, 2017^61^	*ANO10* gene mutation (1)	tCCDCS; anode 3 cm to the right of the inion, cathode over the left motor cortex	Yes	Patient	20 minutes stimulation, 1.5 mA	Decrease in upper limb postural tremor amplitude (accelerometry) No significant change of upper limb dysmetria
Hulst, 2017^40^	SAOA (7), ADCA III (4), SCA14 (3), SCA6 (5), cerebellitis (1)	tDCS; anode 3 cm to the right of the inion, cathode over the right buccinator muscle	Yes	Patients and examiner	22 minutes stimulation, 2 mA	Similar adaptation rates in a force‐field reaching task in the anodal tDCS and sham stimulation groups
John, 2017^59^	SAOA (3), ADCA III (5), cerebellitis (1), SCA6 (2), SCA14 (3)	tDCS; anode 3 cm to the right of the inion, cathode over the right buccinator muscle	Yes	Patients and examiner	25 minutes stimulation, 2 mA	No improvement of grip force control deficits following anodal cerebellar tDCS
Benussi, 2017^63^	SCA2 (5), SCA38 (2), SCA14 (1), FA (1), AOA2 (1), MSA‐C (4), FXTAS (1), SAOA (5)	tDCS; anode 2 cm below the inion, cathode over the right deltoid muscle	Yes	Patients, examiner, and outcome assessor	20 minutes stimulation, 2 mA; 5 days per week for 2 weeks	Decrease in SARA and ICARS scores compared to sham stimulation, specifically the posture and gait and limb coordination items Increased gait speed (8MWT) and improved manual dexterity (9HPT, nondominant hand) compared to sham stimulation Return of CBI after anodal cerebellar tDCS
Benussi, 2018^62^	SCA2 (7), SCA38 (1), SCA14 (1), FA (1), AOA2 (1), MSA‐C (6), SAOA (4)	tDCS; anode 2 cm below the inion, cathode 2 cm below Th11	Yes	Patients, examiner, and outcome assessor	20 minutes stimulation, 2 mA; 5 days per week for 2 weeks; crossover design	Decrease in SARA and ICARS scores compared to sham stimulation, specifically the posture and gait and limb coordination items Increased gait speed (8MWT) and improved manual dexterity (9HPT) compared to sham stimulation Greatest clinical improvement in mildly affected patients Return of CBI after anodal cerebellar tDCS
Dang, 2018^106^	SCA6 (1)	10‐Hz rTMS over the inion (figure‐of‐eight coil)	No	N/A	1,500 pulses per day for 20 sessions (4 weeks)	Decrease in SARA and ICARS scores directly postintervention by 8 and 25 points, respectively, and 11.5 and 30 points, respectively, after 18 months
Manor, 2019^57^	SCA1 (1), SCA2 (1), SCA3 (13), SCA6 (3), SCA8 (1), SCA14 (1)	Neuronavigation‐guided rTMS over the inion and positions 4 cm left and right from the inion (circular coil)	Yes	Patients and outcome assessor	10 pulses over each position every day for 20 days	Similar decreases in SARA score after 1 week Greater percent improvement in SARA score from baseline to 1 month follow‐up compared to the sham group Better performance on the SARA stance item and decreased sway speed and area compared to sham stimulation No differences in 9HPT, Timed Up and Go test, and gait kinematics
Pilloni, 2019^68^	Idiopathic progressive late‐onset ataxia (1)	tDCS; anode on the median line over the cerebellum, cathode on the right shoulder	No	N/A	20 minutes stimulation, 2.5 mA; 5 days per week for 8 weeks; after 2 weeks another 20 sessions	7% faster execution of the 25‐foot walking test 17% faster performance on the Timed Up and Go test 18% (dominant hand) and 19% (nondominant hand) faster execution of the peghole board test Posttreatment motor assessment conducted without the use of a walking aid Decrease in perceived fatigue

OPCA, olivopontocerebellar atrophy; ARCA, autosomal‐recessive cerebellar ataxia; ADCA, autosomal‐dominant cerebellar ataxia; tCCDCS, transcranial cerebellocerebral direct current stimulation; ICARS, International Cooperative Ataxia Rating Scale; AOA2, ataxia with oculomotor apraxia type 2; FXTAS, fragile X–associated tremor/ataxia syndrome; SAOA, sporadic adult‐onset ataxia, SCA, spinocerebellar ataxia; rTMS, repetitive transcranial magnetic stimulation; CBI, cerebellar brain inhibition; tDCS, transcranial direct current stimulation; SARA, Scale for the Assessment and Rating of Ataxia; iTBS, intermittent theta burst stimulation; FTMTRS, Fahn‐Tolosa‐Marin Tremor Rating Scale; FA, Friedreich ataxia; MSA‐C, multiple system atrophy cerebellar type; 9‐HPT, 9‐hole peg test; WT, walking test; N/A, not applicable.

**Table 2:** Overview of studies exploring the therapeutic potential of noninvasive cerebellar stimulation techniques in patients with ET

Study	N	Intervention	Sham	Blinding	Protocol	Results
Gironell, 2002^83^	10	1‐Hz cerebellar rTMS, applied 2 cm below the inion (butterfly coil)	Yes	Patients and outcome assessor	30 trains of 10 seconds with pauses of 30 seconds; crossover design	Improvement of tremor severity 5 minutes after real stimulation, but not 60 minutes after rTMS (FTMTRS and accelerometry)
Popa, 2013^86^	11	Neuronavigation‐guided 1 Hz bilateral cerebellar rTMS (figure‐of‐eight coil)	No	N/A	15 minutes of stimulation of each cerebellar hemisphere during 5 consecutive days	Improvement of tremor severity, writing/drawing, and tremor‐related functional disability at days 5, 12, and 29 Increased functional connectivity between the cerebellum and the motor cortex at day 5
Gironell, 2014^84^	10	tDCS; two cathodes 3 cm left and right from the inion, two anodes over the prefrontal areas	Yes	Patients and outcome assessor	20 minutes of stimulation, 2 mA; 5 days per week for 2 weeks; crossover design	No changes in tremor severity at days 10 and 40 (FTMTRS and accelerometry)
Bologna, 2015^81^	16	Cerebellar cTBS, applied 1 cm below the inion and 3 cm to the right (figure‐of‐eight coil)	Yes	Patients and outcome assessor	Triplets of 50‐Hz stimuli, repeated at 5 Hz for 40 seconds; crossover design	No excitability change in the left motor cortex No change in tremor severity, amplitude, frequency, and reaching movements (FTMTRS and kinematics)
Helvaci Yilmaz, 2016^85^	6	tDCS; two anodes over the dorsolateral prefrontal areas, cathode over the inion	No	N/A	20 minutes of stimulation, 2 mA; 5 days per week for 2 weeks; 1 month later, 5 more sessions every other day	No differences in TETRAS scores between baseline and day 20; lower TETRAS score at day 50 Improvement in ADL scores at days 20 and 50
Shin, 2019^87^	22	1‐Hz cerebellar rTMS, applied 3 cm lateral and 1 cm inferior to the inion over both hemispheres (figure‐of‐eight coil)	Yes	Patients	20 trains of 30 seconds with pauses of 10 seconds over each cerebellar hemisphere; 5 consecutive days	Decrease in total FTMTRS score, including subscales A and B (clinical severity), but not C (daily life activities), immediately and after 4 weeks in both groups without significant group effect

TETRAS, The Essential Tremor Rating Scale; rTMS, repetitive transcranial magnetic stimulation; FTMTRS, Fahn‐Tolosa‐Marin Tremor Rating Scale; cTBS, continuous theta burst stimulation; ADL, activities of dailyliving; N/A, not applicable.

### Degenerative Cerebellar Ataxias

Noninvasive cerebellar stimulation techniques have shown tentatively promising, yet not always consistent, results in patients with degenerative ataxias for whom disease‐modifying and symptomatic treatments are currently lacking. Shiga and colleagues were among the first to touch upon this topic by conducting a sham‐controlled trial in 74 patients with “sporadic or hereditary spinocerebellar degeneration.” They delivered 10 TMS pulses at 100% of maximum stimulator output for 21 consecutive days over both cerebellar hemispheres and the inion. After three weeks of stimulation, gait speed and standing capacities had improved to a greater extent in the intervention group compared to the sham group. Of note, beneficial effects were larger in individuals with pure cerebellar atrophy than in those with olivopontocerebellar atrophy. This improvement was maintained until at least six months after trial cessation when TMS was continued once or twice a week, whereas stimulating once every two weeks quickly led to a return to baseline results.^55^ However, effects on upper limb coordination, speech, and CBI were not addressed, and the authors did not record the precise distribution of ataxia etiologies. Using an identical TMS protocol in a single patient with idiopathic late‐onset cerebellar atrophy, Farzan and colleagues noticed improvements in speech, dysmetria, and tremor, increased gait speed in normal walking and during cognitive dual tasking, and decreased postural sway, paralleled by a reduction of CBI that persisted after six months.^56^ Finally, Manor and colleagues recently published the outcomes of a randomized, double‐blind, sham‐controlled trial in which 20 subjects with various genetically confirmed spinocerebellar ataxias (SCAs) received 20 daily sessions of the aforementioned TMS regimen.^57^ Unfortunately, there were significant baseline differences between the rTMS and sham groups, with the former exhibiting a lower score on the Scale for the Assessment and Rating of Ataxia (SARA), which denotes less severe ataxia, and a faster execution of the Nine‐Hole Peg Test (9HPT). The investigators therefore computed the percent change in each outcome from baseline to follow‐up and reported a larger decrease in SARA score after one month in rTMS‐treated patients, which proved to be due to a better performance on the stance item only. These results were corroborated by more quantitative kinetic postural control tests and the absence of improvement on the 9HPT, suggesting that axial functions may be more susceptible to modification than the appendicular ones.

Cerebellar tDCS might be an exciting new avenue in the treatment of heredodegenerative ataxias that is cheaper, portable, and easier to use compared to TMS devices. In a group of 9 patients with ataxia of various etiologies, anodal tDCS induced a lower amplitude of long‐latency stretch reflexes in the upper limbs, though without amelioration of coordination deficits.^58^ Accordingly, two sham‐controlled studies from the same German group, which included 20 and 14 individuals with heterogeneous cerebellar degenerative disorders, failed to show benefit of anodal tDCS over the right cerebellar hemisphere in a force‐field reaching adaptation task and grip force control task involving the ipsilateral arm.^40^, ^59^ At odds with previous research, these investigators also did not find faster motor adaptation in healthy age‐matched and younger controls after anodal cerebellar tDCS.^40^

In contrast, significant improvements of upper limb postural and action tremor and hypermetria were observed in two SCA2 patients after a protocol involving cerebellar anodal tDCS immediately followed by anodal tDCS over the contralateral motor cortex. These were objectified by spectral analysis of accelerometric data and a decrease of 3 and 3.5 points in the SARA score.^60^ Similarly, the same authors reported a reduction of upper limb postural tremor amplitude in a subject with cerebellar ataxia due to an *ANO10* gene mutation using a modified stimulation technique, with the anode over the right cerebellar hemisphere and the cathode now over the left motor cortex.^61^ Further promising results have come from three randomized, double‐blind, sham‐controlled trials from an Italian group.^62^, ^63^, ^64^ In a heterogeneous sample of nineteen patients with both acquired and hereditary ataxias, Benussi and colleagues showed significantly faster execution of the 8‐meter walk test (8MWT) and 9HPT along with a mean decrease of 1.7 points in the SARA score after a single session of anodal cerebellar tDCS.^64^ In their next study, these investigators applied a two‐week treatment regimen, presumed to generate more cumulative cerebellar excitability changes,^65^ in a group (n = 20) with ataxia of heterogeneous etiologies and showed significant ameliorations in the 8MWT, 9HPT, and SARA score after two weeks of stimulation, the latter still present after three months of follow‐up.^63^ Notably, the reductions in the SARA scores of approximately 3 points roughly correspond to a disease progression of two years in SCAs.^66^ Furthermore, these clinical effects were paralleled by an increase in CBI that also persisted after three months. The significant correlation between symptomatic improvement and return of CBI may imply that a functional restoration of the cerebellocerebral connection is involved in the reduction of ataxia symptoms, but causality cannot be inferred. Patients with less severe ataxia tended to have the largest clinical benefits, probably indicating that the volume of viable cerebellar cortex that can be stimulated is of paramount importance. Interestingly, equally positive effects on ataxia severity, gait speed, manual dexterity, and CBI were obtained after a two‐week treatment with cerebellospinal tDCS in a crossover design involving 21 individuals, again with mixed etiologies.^62^ The results of all these studies are encouraging, but further double‐blind, sham‐controlled, randomized clinical trials are required in more homogeneous cohorts of patients. By corollary, these would yield more robust conclusions per entity and will facilitate decision‐making processes regarding implementation. The varying degree of pathological involvement of the cerebellar nuclei (and cerebellar cortex) per condition may be a pivotal factor, which is not covered in studies with ataxias of heterogeneous etiologies. To this end, a cerebellar tDCS trial involving only SCA3 patients is now ongoing in our center (the Netherlands Trial Register NL7321),^67^ and we are aware of studies focusing on Friedreich ataxia (FA) and the cerebellar type of multiple system atrophy (MSA‐C).

Importantly, an extended schedule comprising 60 daily, remotely supervised tDCS sessions has proven feasible in cerebellar ataxia. This observation may pave the way toward future application of tDCS in a home‐based setting if efficacy can be established on a larger scale.^68^

### Essential Tremor

ET is caused by abnormal oscillatory activity in a network involving the motor cortex, cerebellum, thalamus, and possibly the brainstem.^69^, ^70^ Multiple lines of evidence point to the cerebellum as a key player in the pathophysiology of this disorder. Using a combined electromyography/functional MRI (fMRI) approach, increased tremor‐related activity was found in multiple areas bilaterally in the cerebellum.^71^ Furthermore, ET patients exhibited reduced effective connectivity between the cerebellar cortex and dentate nucleus^72^ and increased functional connectivity between the cerebellar cortex and thalamus.^73^ Postmortem studies revealed pathological changes in all Purkinje cell compartments in the cerebellar cortex (i.e., dendrites, axons, and cell bodies) and reduced gamma‐aminobutyric acid (GABA) levels in the dentate nucleus.^74^, ^75^ The role of GABAergic dysfunction in ET has been further elaborated by nuclear imaging, showing increased 11C‐flumazenil binding in cerebellothalamocortical pathways.^76^ Taken together, these investigations indicate reduced function of the cerebellar cortex in ET, a disinhibition of the deep cerebellar nuclei, and an increased dentate‐thalamo‐cortical drive, which could theoretically result in an abnormal decrease in CBI.

However, two previous CBI studies have reported conflicting outcomes. Hanajima and colleagues found CBI to be significantly reduced compared to healthy controls and therefore proposed involvement of the cerebellar efferent tract in ET.^77^ In contrast, Pinto and colleagues showed normal CBI in younger, more severely affected individuals. Moreover, cerebellar TMS did not result in tremor reset in this study. It was therefore concluded that the tremor oscillator may not reside within the cerebellar cortex, but should be sought in the cerebellar afferent pathway, although the small sample size and interindividual variability in ET may also have contributed to the null finding.^78^ These same investigators later performed TMS examinations in 6 ET patients who had undergone unilateral DBS of the thalamic ventralis intermedius (VIM) nucleus. Relying on the results of their previous work, they ascribed the absence of CBI when DBS was turned off to a thalamic lesion effect (rather than to disease‐specific, intrinsic cerebellothalamocortical tract dysfunction) and showed return of CBI, albeit to a lesser extent than healthy control subjects, in the ON condition.^79^ At first sight, it seems rather puzzling how VIM‐DBS, which is thought to inhibit thalamic neurons through synaptic fatiguing,^80^ can reinstate CBI. A possible explanation would be that DBS selectively suppresses the transmission of pathological signals from the thalamus to the cerebral cortex, leaving the physiological signals along this tract unaffected.

Over the last years, various interventional studies have been conducted in ET using cerebellar 1‐Hz rTMS, cTBS, cathodal tDCS, and tACS.^81^, ^82^, ^83^, ^84^, ^85^, ^86^, ^87^ Unlike in healthy controls, one session of cTBS over the right cerebellar hemisphere did not diminish contralateral motor cortex excitability in 16 ET patients, nor did it improve tremor severity, amplitude, frequency, or the kinematics of reaching movements.^81^ Conversely, a significant, but transient, clinical antitremor effect lasting 5 to 60 minutes was noticed after a single bout of 1‐Hz rTMS over the midline cerebellum.^83^ An open‐label trial investigated the efficacy of a 5‐day course of bilateral cerebellar 1‐Hz rTMS and reported a significant improvement of tremor severity, writing/drawing tasks, and tremor‐related functional disability that was still present after 12 and 29 days. Hence, it is possible that multiple rounds of stimulation also induce cumulative effects in ET.^86^ Furthermore, using resting‐state fMRI, these authors showed that the functional connectivity between the cerebellum and motor cortex, which was severely reduced at baseline compared to healthy age‐matched controls, increased significantly after five days of cerebellar rTMS. Interestingly, the degree of recovery of the functional connectivity on the fifth day was a predictor of tremor severity at day 12. A drawback of this study, however, was the absence of a sham control group.^86^ Finally, the efficacy of five daily sessions of low‐frequency bilateral cerebellar rTMS as add‐on treatment has been investigated recently in a single‐blind, randomized, sham‐controlled study involving 22 patients who still exhibited a troublesome tremor despite using propranolol and/or clonazepam. Both groups displayed a significant decrease in total Fahn‐Tolosa‐Marin Tremor Rating Scale (FTMTRS) score and subscale scores immediately and after four weeks. Importantly, there was no significant group effect and no change in activities of daily living (ADL).^87^

Ten consecutive sessions of bilateral cathodal cerebellar tDCS—also aimed at decreasing Purkinje cell excitability—failed to produce any clinical benefits in ET patients.^84^ Last, a second study with fewer patients and no control group suggested an improvement in ADL and tremor severity after 15 sessions of cathodal cerebellar tDCS.^85^ However, the methodological quality of the latter, as assessed by the PEDro score, was found to be the lowest of the articles discussed in a recent systematic review.^88^

Given its potential to interfere with endogenous brain oscillations, cerebellar tACS is a particularly useful tool to investigate the pathophysiology of tremor. Cerebellar tACS at tremor frequency was able to entrain the phase of the ongoing tremor in patients with Parkinson's disease (PD) and ET, suggesting that the cerebellothalamocortical tract is either part of or connected to the tremor oscillator. Interestingly, entrainment was stronger in patients with a lower frequency tolerance, a newly introduced measure that denotes the range of frequencies over which a tremor is stable.^82^ This finding indicates that a finely tuned set of oscillators (with a narrower frequency tolerance) is more susceptible to interference than a loosely tuned circuit (with a broader frequency tolerance). Future studies may also determine the characteristics of dystonic tremor in this context.

## Discussion and Future Perspectives

Although the anatomical connections between the cerebellum and contralateral motor cortex were described already in the 1960s in animal experiments, some time had elapsed before novel technological interventions allowed the functional exploitation of this tract in humans. The emergence of CBI—an indirect, quantifiable measure of cerebellar excitability—has enriched our understanding of the underlying neural basis of a number of intricate motor tasks in which the cerebellum is implicated.^9^, ^10^, ^11^, ^12^, ^13^ In neurological diseases, it represents a marker that may provide evidence of a functionally or structurally compromised cerebellothalamocortical pathway and therefore deserves to be utilized more frequently as an outcome measure in future cerebellar stimulation trials. Moreover, modulation of cerebellar excitability and connectivity with other brain regions by rTMS or tDCS is a burgeoning area of research that seeks to provide novel insights into the normal functioning of the cerebellum as a node amid various neural networks. The application of noninvasive stimulation modalities in several movement disorders and psychiatric diseases aims to shed new light on the specific role of the cerebellum in their frequently complex pathogenesis and may, in certain cases, also prove to be of therapeutic benefit.^54^, ^89^

Some noteworthy observations of the studies we have reviewed need to be highlighted. First of all, it is important to realize that their methods differed to a great extent with respect to the number of sessions, specific mode of stimulation, and precise placement of the TMS coil or tDCS electrodes. Nevertheless, several investigations in healthy elderly individuals suggest that cerebellar anodal tDCS may be an effective approach to enhance postural control, especially when applied in combination with postural training, and overcome age‐related declines in motor learning.^44^, ^45^, ^46^ In addition, encouraging results of this stimulation technique have also been reported in patients with degenerative cerebellar ataxias, although the scarcity of adequately powered, randomized, double‐blind, sham‐controlled trials and lack of etiological homogeneity in these studies are important shortcomings that need to be appreciated. Yet, multiple rounds of stimulation have repeatedly been shown to mitigate ataxia and induce functional improvement, particularly when medial cerebellar structures were targeted, possibly hinting toward induction of synaptic plasticity.^62^, ^63^, ^68^ By contrast, the effects of a single bout that involves one of the cerebellar hemispheres appear to be less consistent.^40^, ^58^, ^59^, ^60^, ^61^ Second, CBI seems to be reduced in degenerative cerebellar diseases, but can potentially return into the physiological range after multiple sessions of anodal cerebellar or cerebellospinal tDCS. Interestingly, the significant correlation with symptomatic improvement in these studies likely reinforces the direct relevance of the CBI concept in ataxia of degenerative origin, although causality can of course not definitively be inferred.^62^, ^63^ Third, opposing results regarding CBI and the modulation of cerebellar activity have been reported in patients with ET, possibly reflecting the clinical and pathological heterogeneity of this entity.^90^, ^91^ Still, repeated sessions of 1‐Hz cerebellar rTMS (albeit in a single open‐label study) seem to be more effective than cathodal tDCS or cTBS,^81^, ^84^, ^86^ and partial restoration of the cerebellothalamocortical pathway may be realized by virtue of thalamic VIM‐DBS, which is associated with reduction of tremor severity.^79^ However, given the small number of patients included and lack of well‐designed studies that have reproduced these results, the findings should be interpreted with caution. Furthermore, the value of cerebellar rTMS as add‐on therapy to the regular, first‐line drugs has been questioned recently.^87^

In addition to its application in degenerative ataxias and ET, the usefulness of noninvasive cerebellar stimulation techniques has also been examined in individuals with cerebellar ataxia resulting from posterior circulation stroke.^92^, ^93^, ^94^, ^95^ The latter may be considered a third model of cerebellar dysfunction, exemplifying an acute, static cerebellar insult. We have not discussed these investigations here, but a description of their results can be found in Supporting Information Table S1. In brief, the designs of the few studies that have been performed in stroke‐related ataxia were highly heterogeneous in terms of stimulation location (over the injured vs. the healthy cerebellar hemisphere), time frame (acute vs. chronic stroke), duration of stimulation (1, 5, 10, and 14 sessions), and stimulation mode (aimed to increase or decrease the excitability of cerebellar cortical neurons).^92^, ^93^, ^94^, ^95^ This precludes a robust conclusion on the direction of effect of noninvasive cerebellar stimulation in cerebellar stroke. Randomized trials with harmonized stimulation targets and protocols are clearly needed in this area. Of note, patients with middle cerebral artery stroke showed improvement in gait and balance functions after contralateral cerebellar iTBS.^96^

A general remark pertaining to the bulk of studies discussed here concerns the lack of cerebellocerebral connectivity measurements (before and) after noninvasive cerebellar stimulation, which hampers any correlation with clinical changes and thus limits the understanding of the underlying neurophysiological processes. Furthermore, some critical reflections should be made regarding the (sometimes) inconsistent interstudy CBI findings. First, as opposed to the aforementioned investigations in degenerative cerebellar disorders, CBI could be elicited normally in six patients with stroke‐related ataxia and in one with idiopathic late‐onset cerebellar atrophy, and moderate clinical improvement was paralleled by a decrease, rather than increase, of CBI after cerebellar stimulation in these cases.^56^, ^92^ It may thus be argued that the magnitude of cerebellocerebral connectivity and its alteration following stimulation depend on the specific etiology and course of cerebellar pathology, reflecting the variable involvement of cerebellar components and underlying neurophysiological processes in different disorders. Second, this and other observations raise the question of which cerebellar structure becomes primarily modulated by noninvasive stimulation. The figure depicts the commonly held view, pointing to the Purkinje cells in the cerebellar cortex. A number of studies reviewed here, however, report CBI results that are discordant with these assumptions. Accordingly, the situation seems to be much more complex, probably relating to the complicated cerebellar cytoarchitecture and folding pattern as compared to the cerebral cortex,^30^ and the figure should merely be considered a simplified model. Technical considerations that may affect CBI data include the degree to which the TMS coil fits the participant's head and potential coactivation of the brainstem corticospinal tract when using high‐intensity cerebellar conditioning stimuli.^97^, ^98^ Third, stimulation modes known to have opposing effects on cerebellar excitability sometimes induced similar clinical results. Analogous to DBS,^99^ which was initially thought to exert its effects simply by local inhibition of the target structure, it is readily conceivable that cerebellar noninvasive stimulation techniques not just act via a single unifying mechanism of local inhibition or excitation, but rather in multiple ways influencing a whole network.^100^, ^101^, ^102^, ^103^ Fourth, other possible explanations for the sometimes contradictory results may include differences in the disease duration and/or stage of participants, the generally small number of patients, heterogeneity of neuropathology in spite of apparently similar phenotypes, and differences in cerebral resilience and compensation dynamics related to a static lesion or progressive disorder.

The next challenges regarding cerebellar stimulation in degenerative ataxias lie in investigating homogeneous cohorts in terms of (genetic) etiology, exploring the effect and optimal timing of a follow‐up stimulation session, and studying the added effect to intensive training interventions. Future investigations in ET would mainly benefit from including larger numbers of patients exhibiting a similar clinical phenotype and comparable disease severity. The importance of “deeper phenotyping of ET” and including more homogeneous populations in intervention studies was endorsed by the International Parkinson and Movement Disorder Society, which led to the recent publication of a consensus statement on tremor classification.^104^

Besides the clinical effects on the severity of ataxia, tremor, or cognitive dysfunction, measures of cerebellocerebral connectivity, such as CBI, should be determined more regularly to better characterize the neurophysiological substrate of clinical changes after cerebellar stimulation. Last, the addition of imaging techniques and other neurophysiological markers of cerebellar dysfunction may also prove to be a valuable strategy to enhance our understanding of the mechanisms underlying cerebellar stimulation.

## Author Roles

(1) Research Project: A. Conception, B. Organization, C. Execution;

(2) Manuscript Preparation: A. Writing of the First Draft, B. Review and Critique.

R.P.P.W.M.M.: 1A, 1B, 1C, 2A, 2B

R.C.G.H.: 2B

B.P.C.v.d.W.: 2B

## Financial Disclosures

R.C.G.H. serves on the clinical advisory board of Cadent Therapeutics and received honoraria from AbbVie. He receives research support from the Netherlands Organization for Scientific Research and the Michael J. Fox Foundation. B.P.C.v.d.W. receives research support from ZonMw, Hersenstichting, Gossweiler Foundation, Radboud University Medical Center, and Bioblast Pharma and receives royalties from BSL–Springer Nature.

## Supporting information


**Appendix S1**: Supplementary Material
