# Supplementary material for: The role of the cerebellum in degenerative ataxias and essential tremor: Insights from noninvasive modulation of cerebellar activity
Source: Mov Disord. 2019 Dec 10;35(2):215–27. doi: 10.1002/mds.27919 (PMC7027854; doi:10.1002/mds.27919)
Supplement: Supplementary file 1 — Appendix S1: Supplementary Material [file MDS-35-215-s001.docx]

**Supplementary table 1.** Overview of studies investigating the therapeutic benefits of non-invasive cerebellar stimulation techniques in patients with cerebellar ataxia resulting from posterior circulation stroke.

| **Study** | **Etiology (number)** | **Intervention** | **Sham** | **Blinding** | **Protocol** | **Results** |
| --- | --- | --- | --- | --- | --- | --- |
| ***Kim, 2014^1^*** | Posterior circulation ischemic stroke < 3 months (32) | 1 Hz cerebellar rTMS, applied 2 cm lateral to the midline on the ataxic side and 2 cm below the inion (figure-of-eight coil) | Yes | Patients and outcome assessors | 15 minutes stimulation per day for 5 days | - Increased gait speed (10-meter walk) and BBS in both the rTMS and sham stimulation group, according to the authors more pronounced after real stimulation |
| ***Bonni, 2014^2^*** | Posterior circulation ischemic stroke (3) or hemorrhagic infarction (3) | Cerebellar iTBS, applied 3 cm lateral to the midline on the ataxic side and 1 cm below the inion (figure-of-eight coil) | No | Outcome assessor (point in time) | Per day 600 iTBS pulses during 190 s;  5 days per week for 2 weeks | - Reduction of CBI after cerebellar iTBS - Moderate reduction of MICARS total score, specifically the posture and gait items |
| ***Cury, 2015^3^*** | Ischemic stroke in the right cerebellar hemisphere (1) | Neuronavigation-guided 1 Hz rTMS over the left dentate nucleus (double-cone coil) | Yes | Patient and outcome assessor | 25 minutes rTMS; single session | - Improvement of cerebellar ataxia (SARA score minus 8.5 points) and tremor (37% reduction on FTMTRS), but not dystonia after real rTMS |
| ***Buard, 2018^4^*** | Ischemic stroke in the left cerebellar hemisphere 5 years earlier requiring hemispherectomy (1) | 1 Hz cerebellar rTMS, applied 3 cm to the right and 1 cm below the inion (figure-of-eight coil) | No | N/A | 15 minutes stimulation per day for 14 days | - Sensory improvements on the affected side - Easier transfers to and from wheelchair, as reported by a caregiver - Improvement on Finger-Thumb Opposition task, (seated) trunk control, and verbal fluency and recall items on the Montreal Cognitive Assessment - No differences in Rivermead Mobility Index, Stroke Rehabilitation Assessment of Movement, and Tinetti test |

rTMS = repetitive transcranial magnetic stimulation, BBS = Berg Balance Scale, iTBS = intermittent theta burst stimulation, CBI = cerebellar brain inhibition, MICARS = Modified International Cooperative Ataxia Rating Scale, SARA = Scale for the Assessment and Rating of Ataxia, FTMTRS = Fahn-Tolosa-Marin Tremor Rating Scale.

**References**

1. Kim WS, Jung SH, Oh MK, Min YS, Lim JY, Paik NJ. Effect of repetitive transcranial magnetic stimulation over the cerebellum on patients with ataxia after posterior circulation stroke: A pilot study. J Rehabil Med 2014;46(5):418-423.

2. Bonni S, Ponzo V, Caltagirone C, Koch G. Cerebellar theta burst stimulation in stroke patients with ataxia. Funct Neurol 2014;29(1):41-45.

3. Cury RG, Teixeira MJ, Galhardoni R, et al. Neuronavigation-guided transcranial magnetic stimulation of the dentate nucleus improves cerebellar ataxia: A sham-controlled, double-blind n = 1 study. Parkinsonism Relat Disord 2015;21(8):999-1001.

4. Buard I, Berliner JM, Kluger BM. Low Frequency repetitive Transcranial Magnetic Stimulation: Potential role in treatment of patients with hemispheric cerebellar strokes. Brain Stimul 2018;11(3):653-655.
